# An Obscure Multiple Joint Disease of Childhood

**Published:** 1905-03-04

**Authors:** 


### Hospital Clinics.

### AN OBSCURE MULTIPLE JOINT DISEASE OF CHILDHOOD.

Dr. Hale White, in a recent lecture at Guy's Hospital, demonstrated a rare disease first described by Dr. Still in 1896. Dr. Hale White's patient was five years old, and came under observation on account of swelling of many joints, and general enlargement of lymphatic glands. He had suffered a similar illness three years before, and showed great swelling of every observable joint from the smallest to the largest, with the exception of the articulations of the jaw and of the clavicle and scapula. In addition he had enlarged, discrete, movable lymphatic glands in the neck, axillae, and groins, and a moderate enlargement of the spleen. His heart was perfectly healthy.

In discussing the diagnosis the lecturer observed that at first sight the natural conclusion would be that the boy was suffering from osteo-arthritis. But the condition was not osteo-arthritis; for careful examination, confirmed by radiograms, proved that there was no bony overgrowth, nor was there any creaking of the joints on movement, while the remarkable symmetry of the swellings was unlike the irregular lipping to be found in osteo-arthritis. Lastly, there was the lymphatic enlargement which finds no parallel in osteo-arthritis; while the youth of the child, whose age when the disease first appeared was far below that at which juvenile osteo-arthritis usually declares itself, was another argument against the osteo-arthritic nature of the ailment.

Dismissing osteo-arthritis, Dr. Hale White dwelt on the possibility of the condition being a chronic lesion of rheumatism. This he also dismissed, on the score that this child had never shown any of the recognised lesions of rheumatism in childhood, viz. endo-carditis, subcutaneous nodules, tonsillitis or erythemata, while at the same time chronic disability of joints as the result of the true rheumatic process is almost unknown.

The following further possible causes of chronic joint disease were mentioned only to be dismissed. Syphilis, dismissible on the score that its commonest lesion in childhood, epiphysitis, is a lesion of the first year, and that the rare later type of congenital syphilitic joint disease is of the pattern of osteo-arthritis, and thus did not fit the facts. Chronic pyæmia on the ground that the joints were so universally involved. Gonorrhœa and hæmophilia for a similar reason.

Turning to the ætiology of the disease, Dr. White drew attention to the work of Drs. Edsell and Lavenson published in the "American Journal of Medical Sciences," who devoted themselves to the discovery whether or not such patients as these reacted to injections of tuberculin. It was found that the patient they were observing did so react on more than one occasion. Some of the enlarged glands were then removed and submitted to microscopical examination, but no tubercle bacilli could be detected. Yet an emulsion made with the glands revealed bacilli identical in appearance with the tubercle bacillus, but inoculation of rabbits with the emulsion was entirely negative.

The ætiology therefore remains obscure. We may remark that it is common knowledge that chronic glandular enlargement believed to be lymphadenomatous may discredit the diagnosis by suppurating after the lapse of many months or even of years, and it seems not unlikely, on a general view of the facts, that this condition may prove to depend upon a tuberculous infection of very low virulence. At the last meeting of the Society for the Study of Diseases in Children two cases of Still's disease were shown, and in the course of a discussion which followed, a similar case was referred to in which a definite reaction was obtained with tuberculin; but it is noteworthy that the reaction in this instance did not appear to affect either the enlarged glands or affected joints.

